# Mechanisms of promiscuity among drug metabolizing enzymes and drug transporters

**DOI:** 10.1111/febs.15116

**Published:** 2019-11-12

**Authors:** William M. Atkins

**Affiliations:** ^1^ Department of Medicinal Chemistry University of Washington Seattle WA USA

**Keywords:** conformational selection, detoxication enzyme, enzyme promiscuity, induced fit, stochastic enzymes

## Abstract

Detoxication, or ‘drug‐metabolizing’, enzymes and drug transporters exhibit remarkable substrate promiscuity and catalytic promiscuity. In contrast to substrate‐specific enzymes that participate in defined metabolic pathways, individual detoxication enzymes must cope with substrates of vast structural diversity, including previously unencountered environmental toxins. Presumably, evolution selects for a balance of ‘adequate’ *k*_cat_/*K*_M_ values for a wide range of substrates, rather than optimizing *k*_cat_/*K*_M_ for any individual substrate. However, the structural, energetic, and metabolic properties that achieve this balance, and hence optimize detoxication, are not well understood. Two features of detoxication enzymes that are frequently cited as contributions to promiscuity include the exploitation of highly reactive versatile cofactors, or cosubstrates, and a high degree of flexibility within the protein structure. This review examines these intuitive mechanisms in detail and clarifies the contributions of the classic ligand binding models ‘induced fit’ (IF) and ‘conformational selection’ (CS) to substrate promiscuity. The available literature data for drug metabolizing enzymes and transporters suggest that IF is exploited by these promiscuous detoxication enzymes, as it is with substrate‐specific enzymes, but the detoxication enzymes uniquely exploit ‘IFs’ to retain a wide range of substrates at their active sites. In contrast, whereas CS provides no catalytic advantage to substrate‐specific enzymes, promiscuous enzymes may uniquely exploit it to recruit a wide range of substrates. The combination of CS and IF, for recruitment and retention of substrates, can potentially optimize the promiscuity of drug metabolizing enzymes and drug transporters.

## Graphical abstract

Drug metabolizing enzymes and drug transporters exhibit remarkable substrate promiscuity that is critical for their function as detoxication enzymes. These enzymes exploit mechanistic variations of substrate‐specific enzymes in order to achieve their promiscuity. For example, drug metabolizing enzymes utilize the same cosubstrates substrate specific enzymes, but they decouple substrate binding from cosubstrate activation. Drug metabolizing enzymes also exploit conformational flexibility in novel ways to enhance their promiscuity.

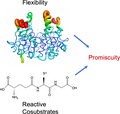

**Abbreviations**
AO: aldehyde oxidase
CS: conformational selection
CYP: cytochrome P450
EH: epoxide hydrolase
FMO: flavin monooxygenase
GSH: glutathione
GST: glutathione transferase
H/DX MS: hydrogen/deuterium exchange mass spectrometry
IF: induced fit
mEH: microsomal epoxide hydrolase
P‐gp: P‐glycoprotein
SULT: sulfotransferase
UDPGA: uridine 5'‐diphosphoglucuronic acid
UGT: uridine 5′‐diphospho‐glucuronosyl transferase

## Overview

Detoxication enzymes and transporters play a critical role in drug metabolism and drug–drug interactions. In fact, some of these enzymes are colloquially referred to as 'drug‐metabolizing' enzymes and ‘drug transporters’, but it is useful to emphasize that they have evolved to play a much broader role in the detoxication of environmental compounds. Drug metabolism is the 'collateral damage' that is incurred by the remarkable promiscuity that these enzymes exhibit, which is critical for their detoxication function. Included among this cohort are cytochrome P450s (CYPs), UDP‐glucuronosyl transferases (UGTs), glutathione transferases (GSTs), flavin monooxygenases (FMOs), epoxide hydrolases (EHs), aldehyde oxidases (AOs), efflux transporters such as P‐glycoprotein (P‐gp), and others. Many of these enzymes exhibit both substrate promiscuity, which is the ability to bind and perform the same metabolic reaction on structurally unrelated substrates, and catalytic promiscuity, which is the ability to catalyze many types of chemical reactions on chemically distinct functional groups with significantly different local transition states as defined previously 1, 2, 3, 4, 5, 6. Because the detoxication efflux transporters do not chemically alter the molecules they export, only ‘substrate promiscuity’ is relevant to their function. Previous reviews have suggested that drug metabolizing enzymes are not ‘promiscuous’ but rather are ‘multispecific’ or ‘substrate ambiguous’, or they have ‘broad specificity’ 1, 2, 3, 4, 5, 6. This distinction was based on a definition of promiscuity that was limited to metabolism of a noncognate substrate by a substrate‐specific enzyme. I previously asserted that enzymes with no cognate substrate are uniquely promiscuous; it is their job to be promiscuous 5. So, I apply the term to drug metabolizing enzymes and transporters.

Arguably, detoxication enzymes are fundamentally different from 'normal' enzymes: Their functional value can be optimal when they exhibit only modest catalytic rate enhancement as long as it includes a wide range of substrates, including substrates that have been previously unencountered, rather than optimizing rates with any individual substrate. This difference demands consideration of the molecular properties that promote promiscuity. The properties that confer promiscuity to drug metabolizing enzymes are likely to be different from the properties that provide the optimal rate enhancements with any single substrate. However, this premise is clouded by our inadequate understanding of the relationship between specificity or promiscuity and rate enhancement. Examples exist for mutations that increase the specificity for the cognate substrate and for a general increase in flux for several substrates upon single mutation, where ‘flux’ is considered here as the rate of product formation at any specific concentration of substrate 7, 8. Other examples suggest that evolution can simultaneously improve more than one catalytic function of a promiscuous template enzyme 8. So, we are still learning about the relationship between specificity or promiscuity and catalytic rate enhancement.

A great deal of attention has focused on the evolutionary importance of the other types of ‘promiscuity’, wherein substrate‐selective enzymes or their mutants have the ability to catalyze reactions with noncognate substrates 1, 2, 3, 4, 6, 7, 8. It is clear from many studies that divergent evolution of new function from a fixed pool of dynamic protein scaffolds is facilitated by such mutations that modestly increase the promiscuity of a substrate‐selective enzyme, which becomes a ‘generalist’. The promiscuous generalist, mutant or wild‐type, can serve as a template for facile evolutionary optimization of the new substrate‐specific activity. Promiscuous templates obtained from substrate‐specific enzymes are evolutionary intermediates, and the cost of ‘mutational noise’ in the sequence of the proteome that generates templates is useful for adaptation. An interesting aspect of the generalist‐to‐specialist paradigm is that promiscuous detoxication enzymes could easily adapt to environments with changing toxins and remain promiscuous within a different realm of chemical space. Mannervik *et al*. have elegantly shown that some detoxication enzymes can be easily converted via directed evolution *in vitro* to equally promiscuous enzymes with orthogonal substrate promiscuity 9, 10. So detoxication enzymes and transporters may undergo generalist‐to‐generalist evolution to detoxify compounds that occupy different regions of chemical space, but which would require retention of the properties that afford promiscuity. What are these properties? Detoxication enzymes likely represent the result of evolutionary pressure to maintain, and optimize, promiscuity, rather than to eliminate it. Thus, the limits of promiscuity approached by a theoretically ‘perfect’ detoxication enzyme represent the approach to promiscuous evolutionary endpoints rather than intermediates. Detoxication enzymes potentially represent the benchmark for the limits of substrate promiscuity, so consideration of the mechanisms by which they achieve their promiscuity is instructive.

The suggestion that detoxication enzymes are quantitatively more promiscuous than their structurally related substrate‐specific homologs is supported by application of a quantitative index in a few cases, based on relative *k*_cat_/*K*_M_ values across a series of substrates and normalized to account for the structural diversity within the substrate series 11. This promiscuity index defines ‘*J*‐values’ that are a relative measure of the ability of closely related enzymes to metabolize a range of substrates without preference for any specific one. The resulting scale of promiscuity *J*‐values ranges from 0 (perfect specificity for one substrate in the series) to 1 (no preference for any substrate over another within the series). Drug metabolizing enzymes have *J*‐values > 0.7, whereas their corresponding substrate‐specific homologs have *J*‐values between 0.3 and 0.6 11, 12. Similarly, promiscuous proteases vs. specific proteases have *J*‐values of ~ 0.8 and near 0, respectively, in accordance with their physiological functions 11. Other methods to quantify promiscuity have been developed but not applied directly to compare drug metabolizing enzymes 13. It is worth noting that even enzymes considered to be highly substrate‐specific can modestly catalyze reactions with noncognate substrates at high concentrations, and all enzymes are capable of some promiscuous behavior. Regardless of whether quantitative indices are applied, the high level of substrate promiscuity among detoxication enzymes and transporters is undeniable. Because the mechanisms of substrate promiscuity among detoxication enzymes are not well established, some of this article includes prospective, even speculative, scenarios intended to prompt further work in this area.

## Substrate specificity as a contrast

### Structural and energetic bases of substrate specificity

In order to consider the possible attributes of an enzyme that optimizes promiscuity, it is useful to consider first some properties that contribute to substrate specificity, which are well established and understood. Benchmarks for the limits of substrate specificity and catalytic perfection are rooted in structural, kinetic, and energetic considerations. Energetic and kinetic criteria for optimization of substrate‐specific enzymes are based on *k*_cat_ or *k*_cat_/*K*_M_, or ‘flux’ of substrate to product. For example, classic work of Knowles & Albery, and others, describes the evolutionary perfection of enzymes that starts with ‘uniform binding’ or equivalent stabilization of substrate complexes, product complexes, and transition states 14, 15. Contrasting models have been considered, but they are still based on flux and *k*_cat_/*K*_M_ as criteria to be optimized 16. In the conceptual framework of Knowles *et al*., further evolution leads to ‘differential’ stabilization of the rate‐limiting transition‐state vs. ground‐state substrate or product complexes. This energetic perspective suggests that substrate‐specific enzymes perfect catalysis by avoiding clear rate‐limiting steps and having nearly equal energy barriers when many steps are involved 17.

Notably, all of these mutational processes that lead to catalytic perfection during evolution are assumed to affect interactions with the cognate substrate on which the enzyme normally acts. It is presumed in analyses of evolutionary processes that the optimal changes in energetic profiles would be those that improve catalysis, either *k*_cat_ or *k*_cat_/*K*_M_, with the specific cognate substrate(s), without considering the interactions with noncognate substrates. These ideas have been amplified and refined by others in the context of promiscuous enzyme templates 7, 18, with the suggestion that evolution of specificity likely accompanies catalytic improvements toward the cognate substrate, and hence flux of specific substrate to specific product.

Structural considerations also reveal mechanisms of substrate specificity. In fact, it might be argued that the structural biology revolution demystified the ‘amazing’ substrate specificity attributed to many enzymes in the infancy of enzymology. As a result of the structural biology revolution of the 1980s–1990s, our understanding of enzyme 'specificity' is reasonably mature. We have learned how enzymes from many structural families can recognize specific substrates with great selectivity compared to close structural substrate analogs, by straightforward exploitation of shape, charge, hydrophobic interactions, and hydrogen bonding complementarity 19, 20, 21, 22, 23, 24, 25. Also, as described in the article by Tawfik and Gruic‐Solvulj in this issue, nature has also exploited mechanisms to explicitly select against noncognate substrates. These mechanisms are outside the scope of this focus on promiscuity, but they further emphasize the wide range of mechanisms by which enzymes achieve specificity. When structural models are combined with theoretical aspects of enzyme catalysis rooted in energetics and kinetics, we achieve a solid understanding of mechanisms that confer enzymes with specificity.

## Mechanisms of promiscuity

In contrast to the devices used by substrate‐specific enzymes, we understand little about the mechanisms by which drug metabolizing enzymes optimize their function. How does evolution optimize promiscuous enzymes in the absence of a defined substrate to define selective pressure? Arguably, it is a more challenging problem to evolve or design an enzyme that efficiently metabolizes many structurally diverse substrates than to design or evolve a highly specific enzyme that only needs to perform a single reaction. Flux of any individual substrate to product is unlikely to be the most important catalytic property for promiscuous detoxification enzymes, so it is difficult to superimpose models for the optimization of substrate‐specific enzymes on detoxication enzymes. Still, an interesting analysis of the detoxication aldose reductase in terms of flux and kinetic parameters has suggested the importance of cofactor binding over substrate binding to one‐way flux 26. This example is a reminder that concepts used to explain highly substrate‐specific flux are useful in some cases, but insufficient to understand completely the promiscuity of detoxication enzymes. Detoxication enzymes must balance flux of substrate to product with versatility.

### Reactive cofactors, cosubstrates, and high‐energy conformations

Three decades ago, Bill Jakoby noted in review articles that the promiscuous detoxication FMOs, sulfotransferases (SULTs), and GSTs stabilize the reactive form of their respective cofactors or cosubstrates in the absence of any substrate 27, 28. Specifically, detoxication FMOs bind the nucleophilic 4a‐hydroperoxy flavin anion and 'wait' for substrates to bind and encounter the reactive flavin. The formation of the reactive hydroperoxyl flavin does not depend on the presence of a substrate. Jakoby noted also that multiple promiscuous SULTs utilize a common electrophilic cosubstrate, 3’‐phospho‐adenosine‐5’‐phosphosulfate (PAPS), to transfer sulfate to many structurally unrelated nucleophiles, but for each individual SULT, many substrates had apparent access to PAPS. Similarly, many GSTs considered to be drug metabolizing enzymes lower the *pK*_a_ of the thiol of bound glutathione (GSH) to ~ 6.7 so that GSH is largely ionized as the nucleophilic thiolate, GS^‐^, at the active site 29. In each case, the cosubstrate is reactive in the absence of substrate and poised to react with any electrophilic or nucleophilic substrate that finds itself nearby. Jakoby noted that the formation of a reactive intermediate form of a cofactor or cosubstrate, independent of substrate binding, was a potential mechanism by which detoxication enzymes could divorce catalytic activity from substrate specificity. Table 1 summarizes a partial list of human drug metabolizing enzymes and their respective cofactors or cosubstrates. The accuracy of Jakoby's initial proposal is underscored by considering more recent results with various GST isoforms. All GSTs considered to be detoxication enzymes have been shown to stabilize the thiolate anion of GS^‐^ relative to the protonated thiol when GSH is bound, via hydrogen bonding to an active tyrosine or serine 30, 31. The importance of this is clarified by considering two GSTs that are nearly identical in their amino acid sequences but perform different biological roles. GSTA1‐1 is the prototypical detoxication enzyme, highly expressed in liver and kidney, and it contributes to drug metabolism, with a *pK*_a_ of bound GSH of ~ 6.8 32, 33. In contrast, the nearly identical GSTA4‐4 is highly substrate‐specific, with a marked preference for long‐chain lipid aldehydes, and it is expressed at very low levels in the liver and kidneys. Many studies suggest GSTA4‐4 plays a specific role in the metabolism of hydroxynonenal 34. In further contrast, GSH bound to GSTA4‐4 remains protonated, with a *pK*_a_ of > 9, and is not poised to react with 'any nearby electrophile'. Presumably, long‐chain lipid aldehydes specifically induce deprotonation of GSH, as suggested by Hubatsch and Mannervik, although this has not been directly demonstrated 35. This example implies that evolution has utilized the ability to couple GSH ionization with substrate binding with a substrate‐selective enzyme but left GSH ionization and substrate binding uncoupled when promiscuity is desired. This frames Jakoby’s initial suggestion that reactive cofactors are indiscriminately presented to substrates in detoxication enzymes.

**Table 1:** Major drug metabolizing enzymes and their cofactors or cosubstrates.

Enzyme family with detoxication functions in humans: examples of isoforms	Cofactor or cosubstrate	Reactive form of cofactor or cosubstrate
CYPs: CYP3A4, CYP3A5, CYP2C9, CYP2C19, CYP2D6, CYP1A2, and others	Hematoporphyrin IX cofactor	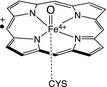
FMOs: FMO1, FMO2, and FMO3	Flavin adenine dinucleotide cofactor	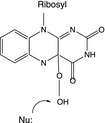
UGTs: UGT1A1, UGT2B7, and others	UDPGA cosubstrate	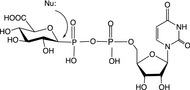
GSTs: GSTA1, GSTA2, GSTM1, GSTM3, and others	GSH cosubstrate	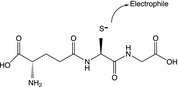
SULTs: SULT1A, SULT1A2, SULT2A1, and others	3′‐Phospho‐adenosine 5′‐phosphosulfate cosubstrate	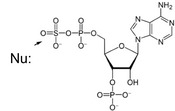
AO: AOX1	Pyranopterin molybdate cofactor	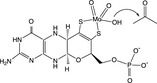

An interesting extension of the reactive cofactor role in substrate promiscuity is the possibility that multiple isoforms of an enzyme with slightly different chemical reactivities of their cofactor could collectively achieve greater promiscuity and afford better protection from the widest range of toxins. This theoretical possibility has been considered with GSTs 36. Specifically, the Bronsted relationship predicts that there is a trade‐off between lowering the *pK*_a_ of GSH to generate more GS^‐^ and decreasing the nucleophilicity of the resulting thiolate 37, 38. In fact, different GST isoforms with different substrate selectivities exhibit different pK_a_s for their bound GSH and this could contribute to the collective substrate promiscuity of GSTs 36. Regardless of this speculative possibility, it is noteworthy that evolution has poised differentially the pK_a_s of these GSTs near the maximum predicted by Bronsted behavior for a range of electrophilic substrates.

#### Cytochrome P450 monooxygenases

The CYPs dominate drug metabolism and provide an impressive level of protection from environmental toxins 39, 40. CYPs certainly fit the initial proposal of Jakoby, wherein the same heme cofactor found in many proteins is uniquely tuned by the canonical CYP fold to stabilize an extraordinarily reactive high‐valent iron‐oxo intermediate, Compound I 41, 42, 43. Both substrate‐selective CYPs, which are critical for the biosynthesis of endogenous components of homeostasis or metabolism of specific carbon sources, and drug‐metabolizing CYPs utilize Compound I to conduct an extraordinary range of chemical oxidations, including hydrocarbon hydroxylation, olefin epoxidation, C‐C bond cleavage, and heteroatom dealkylation. Individual CYP isoforms are capable of all of these types of metabolism.

In fact, in many cases several catalytic reactions are performed by a single CYP on the same substrate. A drug or toxin with multiple functional groups or sterically unhindered carbon atoms may yield a heterogeneous mixture of metabolites from a single CYP isoform. This ‘product promiscuity’ is an additional affirmation of Jakoby’s initial mechanistic insight; detoxication enzymes generate highly reactive cofactors that wait for a substrate to ‘bump into them’, without orchestrating a specific orientation for a transition state. The wealth of intramolecular kinetic isotope effects that lead to metabolic switching (isotope‐dependent change in regioselectivity) is observed with CYP reactions, and the distinct lack of regioselectivity when multiple oxidizable sites are present within a single substrate is consistent with highly dynamic substrate complexes that allow the substrate to sample multiple orientations and allow multiple possible sites of oxidation to bump into the reactive cofactor 44, 45, 46. Such dynamic substrate enzyme complexes seem at odds with the classic views of enzyme optimization for substrate‐specific enzymes that include ‘differential binding’ of transition state vs. ground states 14, 15, and more recent analyses that reemphasize the importance of preferential binding of transition states via stiff complexes vs. flexible ligand‐free ground states 47. This product promiscuity is a direct result of formation of ‘indiscriminate’ reactive cofactor intermediates utilized by detoxication enzymes to achieve substrate promiscuity, and it amplifies the contrast between substrate‐specific enzymes and detoxication enzymes.

#### Flavin monooxygenases

As noted above, FMOs were among the enzymes considered by Jakoby and they contribute to the oxidation of drugs with nucleophilic heteroatoms including tertiary amines 48, 49, 50. There are five cytosolic human isoforms, of which FMO1 is a fetal liver enzyme that is replaced by the major drug‐metabolizing FMO3 in the adult liver. These FMOs are cytosolic NADPH‐dependent enzymes. An early intriguing observation was that the observed V_max_ for FMOs is independent of the substrate being oxygenated, which presumably prompted Jakoby and others to consider the difference between detoxication enzymes and substrate‐specific enzymes. This substrate independence of rate is paralleled by substrate‐independent formation of the 4a‐hydroperoxy flavin intermediate that reacts with nucleophilic substrates. A distinctly different group of FMOs from prokaryotes, known as Baeyer–Villiger FMOs, uses the 4a‐hydroperoxy flavin intermediate as a nucleophile, wherein the distal oxygen attacks a carbonyl. Apparently, evolution has repurposed this intermediate for various functions, including oxidation of nucleophilic xenobiotics with the drug‐metabolizing FMOs 51.

#### UDP‐glucuronosyl transferases

The hepatic UGTs, individually and collectively, also metabolize an extraordinary range of structurally unrelated drugs 52, 53, 54. The UGTs are complex membrane‐bound enzymes with isoform‐dependent N‐terminal domains that encode different substrate preferences, although each is highly promiscuous. No crystal structures of full‐length UGTs are available, but a structure of the C‐terminal domain from UGT2B7 has clarified the mechanism of specificity for binding to the UGT cofactor 53. UGTs exploit the reactive cofactor uridine 5'‐diphosphoglucuronic acid (UDPGA), which is highly electrophilic at the 1 carbon of the glucuronic acid due to the good phosphate leaving group. Many nucleophilic functional groups on a wide range of substrates react with UPGA within UGT active sites, including alcohols, phenols, alkyl and aryl amines, hydroxyl amines, thiols, and even carbon atoms anionic character. As with the CYPs, UGTs frequently generate multiple products form a single substrate, when multiple nucleophiles are present 52, 54. Clearly, substrates can bind in multiple orientations to a single UGT isoform.

#### Cytosolic sulfotransferases

The cytosolic hepatic SULTs catalyze the sulfation of a wide range of xenobiotic phenols, alcohols, and amines 55, 56, 57. As noted above, the electrophilic cosubstrate PAPS is exploited to transfer a sulfate to acceptor drug substrates. Interestingly, SULT1A1 and SULT1A2 bind PAPS significantly more tightly than acceptor substrates, whose affinity is not affected by the presence or absence of PAPS. A conformational closure of the active site around PAPS provides a selectivity filter of SULT1A1 that accepts small substrates at low concentrations and larger substrates only at high concentrations 56, 57. The reactive cosubstrate is poised to indiscriminately react with acceptors that enter the active site via either conformation. As with CYPs and UGTs, SULT also exhibits product promiscuity 58, 59.

#### Aldehyde oxidase

Aldehyde oxidase performs a wide range of chemical oxidations on structurally diverse substrate aldehydes and aza‐ or oxo‐heterocycles and has recently become appreciated for its contribution to drug metabolism 60, 61. A single isoform, AOX1, is highly expressed in human liver, consistent with its role in detoxication. The AOX structure is a complex homodimer with each subunit having multiple domains with redox‐active cofactors 62, 63. It includes in its structure FAD, a molybdenum pyranopterin cofactor, and iron–sulfur clusters. The molybdenum is used to generate a highly reactive, nucleophilic, hydroxyl intermediate (CoMo) that attacks carbonyl groups or α‐carbons of imines.

#### P‐glycoprotein (ABCB1)

In addition to enzymes that perform metabolic transformations, many efflux transporters contribute to detoxication of environmental toxins and drugs by pumping them out of cells and facilitating their clearance 64, 65, 66. P‐gp, or ATP Binding Cassette transporter B1 (ABCB1), is arguably the most promiscuous transporter known. Despite many attempts to define preferred substrate characteristics, transported substrates share little in common other than being hydrophobic, and P‐gp exhibits a modest preference for substrates with a positive charge. P‐gp utilizes ATP hydrolysis to drive a conformational switch between an inward‐facing ensemble that captures substrates from the membrane, to outward‐facing conformations from which substrates diffuse to the extracellular milieu 66, 67, 68, 69, 70. The structure of P‐gp is complex and includes 12 transmembrane helices that provide binding sites for drugs that diffuse in from the membrane, and two cytosolic nucleotide‐binding domains that each bind and hydrolyze ATP. There is limited detailed structural information about the drug‐binding site although it is expansive and can accommodate very large substrates. Although no chemical transformation occurs on the drug as it is effluxed, the coupling of ATP hydrolysis with the formation of high‐energy outward‐facing conformations that promote diffusive efflux is analogous to the formation of high‐energy reactive cofactors that perform chemical transformations in the true metabolism enzymes. An indiscriminate inward‐facing conformation allows a wide range of drugs or toxins to bind and its conversion to higher energy outward‐facing conformation provides a diffusive pathway to the extracellular space for any toxin that has found its way into the active site, before relaxing to the inward‐facing state.

#### Was Jakoby right?

The examples summarized above indicate the relevance of Jakoby’s early suggestion that highly reactive cofactors are a common device used by promiscuous enzymes. As suggested by Jakoby, recent studies based on systematic and extensive mutagenesis studies with mutants of a bacterial detoxification FMO lead to the conclusions that the role of the protein is only to provide a ‘shell’ for the reactive cofactor, rather than to contribute to interactions with substrates 71.

Despite these examples, the presence of reactive cofactors among detoxication enzymes is not a strictly necessary condition for substrate promiscuity. For example, some microsomal EHs (mEHs) considered to be detoxication enzymes do not use cofactors. The mEHs utilize an active‐site aspartic acid carboxylate to attack the epoxide of a drug or toxin and generate a covalent acylated substrate that is hydrolyzed to regenerate enzyme and free substrate diol 72, 73.

Conversely, many highly substrate‐specific enzymes utilize the same cofactors and cosubstrates as those mentioned above. So, the presence of these cofactors or cosubstrates is not a sufficient condition for substrate promiscuity (a case could be made perhaps that they are necessary for catalytic promiscuity, wherein different reactions with different local transition states can be accommodated by cofactors more readily than the amino acid side chains available in active sites). In summary, despite their prevalence among detoxication enzymes, reactive cofactors or cosubstrates are neither a necessary nor a sufficient condition for substrate promiscuity.

### Conformational plasticity or ‘flexibility’

In addition to the exploitation of reactive cofactors, Jakoby suggested that a second property of detoxication enzymes that contributes to promiscuity is a conformationally flexible active site that can accommodate a wide range of substrates in proximity to the cofactors or cosubstrates 26, 27. Notably, his suggestion predates any crystal structures of detoxication enzymes. Although this is intuitively sensible, it is difficult to quantitatively assess the hypothesis that flexibility yields promiscuity. Few literature examples explicitly define ‘flexibility’ or ‘promiscuity’. Jakoby’s intuitively comfortable suggestion is supported qualitatively by crystallographic structures of detoxication enzymes where different ligands are bound to significantly different conformations of a single enzyme. However, a full understanding would be facilitated by some thermodynamic and kinetic context for flexibility as it relates to promiscuity and specificity. It is also illustrative to examine more closely this intuitive suggestion in the context of the well‐established formalism concerning protein dynamics and ligand interactions for substrate‐specific enzymes and proteins: induced fit (IF) vs. conformational selection 74, 75, 76, or the additional case of the ‘lock and key’ model, in which a rigid preformed active site in the ligand‐free state is complimentary to, and unaffected by, ligand. These limiting case models highlight subtle and interesting possible mechanism by which detoxication enzymes could optimize their promiscuity.

IF refers to the case when a single ‘inactive’ enzyme conformation in the absence of substrate binds to the substrate, which induces a change to an active conformation. This ubiquitous behavior among enzymes provides a source of substrate specificity, as long as only the cognate substrate induces the correct conformational change 77. For IF, the conformational change occurs after binding.

In contrast, conformational selection occurs when an equilibrium of conformations exists in the absence of substrate, which selects one ‘active’ conformation to bind to. For substrate‐specific enzymes, CS has no advantage, and equilibrating states in the absence of ligand do not provide any catalytic or specificity advantage. An equilibrium of states in the absence of substrate, as in CS, is the cost that is paid to maintain the flexibility required for IF, because the nonbinding conformations of the substrate‐free ensemble could interact with noncognate substrates and lead to inhibition, or these conformations would decrease the apparent affinity of substrate for the active conformation. For conformational selection, the conformational change occurs before binding and cannot be advantageous for flux of cognate substrate to product.

It is likely that the vast majority of enzymes exhibit combinations of both behaviors, which are not mutually exclusive. In fact, IF and CS are thermodynamically equivalent, path‐dependent, extremes of a continuum and it may be unrealistic to assign ‘one or the other’ to any individual enzyme. To the extent that these behaviors require flexibility or plasticity, IF and CS are natural starting points for considering the widespread expectation that flexibility correlates with promiscuity.

#### Glutathione transferases, conformational selection, and induced fit

As noted above, two GST isoforms represent benchmarks for high specificity and high promiscuity within a single protein fold and they provide insight into a thermodynamic and kinetic base for promiscuity, which can be interpreted in the context of IF vs. CS. The GST isoform GSTA1‐1 is highly promiscuous, whereas GSTA4‐4 has evolved specifically to clear 4‐hydroxynonenal or long‐chain lipid aldehydes 34. GSTA4‐4 is not a promiscuous detoxication enzyme. The promiscuity *J*‐values for GSTA1‐1 and GSTA4‐4 are 0.79 and 0.43, respectively 11, which is a remarkable difference given their sequence similarity.

**Figure 1:**
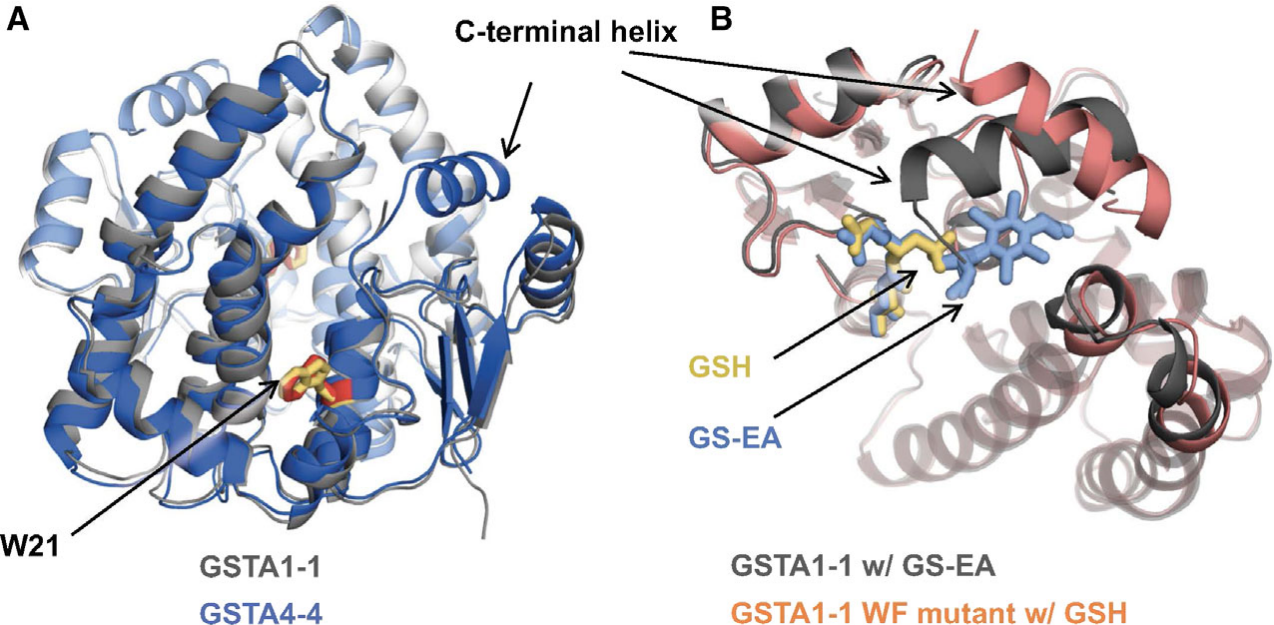
GST structural features. (A) Overlay of GSTA1‐1 and GSTA4‐4 apo structures in gray and blue, respectively (PDB http://www.rcsb.org/pdb/search/structidSearch.do?structureId=1GSD and http://www.rcsb.org/pdb/search/structidSearch.do?structureId=1GUM). GST forms a homodimer; the second subunit is in the background in lighter gray/blue. The C‐terminal helix is flexible and not resolved (not observed here) in most apo A1‐1 structures. Trp21 is conserved in both GSTA1‐1 and GSTA4‐4 and lies at the intrasubunit domain–domain interface remote from the active site (yellow/red, A1‐1/A4‐4). (B) Overlay of GSTA1‐1 with GS‐EA substrate conjugate bound and A1‐1 mutant with GSH bound in which the C‐terminal helix is resolved in one subunit in gray and red, respectively (PDB http://www.rcsb.org/pdb/search/structidSearch.do?structureId=1GSE and http://www.rcsb.org/pdb/search/structidSearch.do?structureId=1EV4). The C‐terminal helix can adopt very different locations when different ligands or substrates are bound.

An obvious difference in protein dynamics between these isoforms lies at their C termini, which contribute to their active sites. The C‐terminal 14 residues of GSTA1‐1 form a mobile helix in the absence of substrates, with low electron density in crystallographic models and poorly resolved NMR peaks (Fig. 1) 78, 79, 80. The active site is analogous to the molten globule states of folded proteins with heterogeneous tertiary contacts. The helix becomes ordered but adopts distinctly different locations along the active site with different substrates or product conjugates. This is a clear, but noncanonical, example of ‘IF’. In effect, with GSTA1‐1 different substrates can induce different conformations of the active site, and different conformations are catalytically competent, which might be described as ‘IFs’. In contrast, GSTA4‐4 exhibits the behavior originally described as ‘lock and key’ binding. The C‐terminal helix is well ordered in the absence of ligands and forms one wall of the long hydrophobic active site and a substrate analog binds with no detectable rearrangement of the C‐terminal helix of GSTA4‐4 81, 82. Interestingly, based on hydrogen/deuterium exchange mass spectrometry (H/DX MS) and time‐resolved fluorescence, the differences in dynamics of GSTA1‐1 and A4‐4 are not completely localized to the C terminus. The GSTA1‐1 exhibits increased dynamics based on H/D exchange mass spectrometry throughout the entire structure despite nearly superimposable crystal structures, and the single Trp‐21 at the domain interface of GSTA1‐1 exhibits increased conformational heterogeneity compared to the homologous Trp in GSTA4‐4 83. Furthermore, a series of ‘swap’ mutants of GSTA1‐1 with increasing numbers of residues from the GSTA4‐4 active site has been constructed and the mutants exhibit a range of promiscuity *J*‐values. One mutant, called the GIMFhelix, has ~ 600‐fold greater activity toward HNE than GSTA1‐1 does, and its crystal structure reveals a highly ordered immobile C‐terminal helix 84.

**Figure 2:**
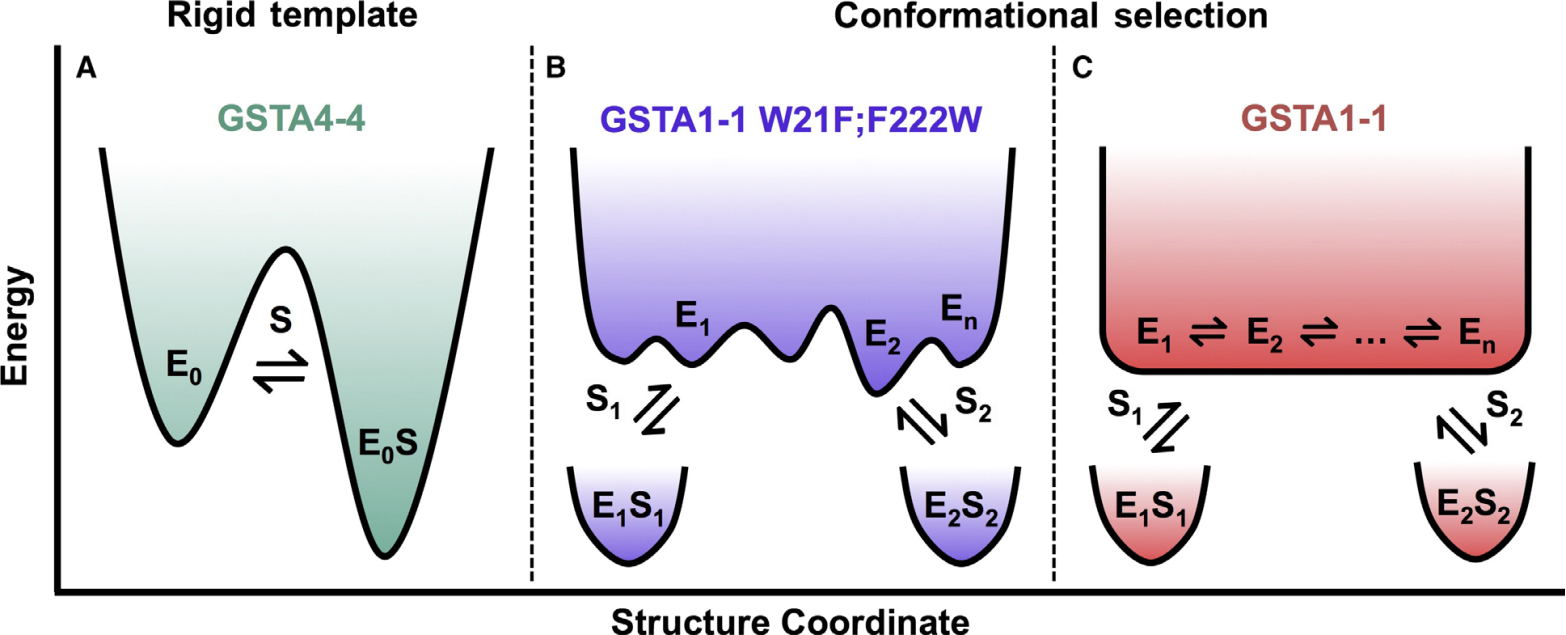
Schematized free energy landscapes for substrate‐specific ligand‐free wild‐type GSTA4‐4 (a), a mutant GSTA1‐1 with intermediate promiscuity that exhibits pre‐steady‐state lags or bursts (b), and promiscuous wild‐type GSTA1‐1 (c). The conformational landscape of GSTA1‐1 has minimal energy barriers to rearrangement, so there is minimal kinetic cost for the conformational selection wherein different substrates select different conformations. After binding S1 or S2, further conformational change occurs (IF). The mutant GSTA1‐1 W21F;F222W (b) has modest energy barriers that add kinetic lags or burst in steady‐state catalytic experiments, but they are not sufficiently large to prevent conformational heterogeneity. In both b and c, different substrates select different conformations.

The wild‐type GSTA1‐1 and A4‐4, along with this series of mutants, were used to quantitatively asses the hypothesis that flexibility confers promiscuity. Fortuitously, differential scanning calorimetry (DSC) revealed a low‐temperature reversible transition, prior to the main unfolding, with GSTA1‐1 that is absent with GSTA4‐4. This DSC transition was assigned to temperature‐dependent conformational flexibility of the C terminus of GSTA1‐1, based on several criteria 85, 86. The DSC behaviors of the wild‐type GSTA1‐1 and GSTA4‐4, along with the series of mutants that altered the promiscuity *J*‐values, were analyzed with a barrierless transition model that provides a surrogate free energy landscape for the low‐temperature conformational transition of each GSTA variant 87. The resulting energy/enthalpy landscapes ranged from smooth and wide, with no significant energy barriers between enthalpic states, to rough landscapes, wherein deep energy wells restricted conformational sampling in the relevant temperature range (Fig. 2) 86. In effect, the analysis provided a quantitative comparison of the ‘flexibility’ of this series of proteins, based on a thermodynamic formalism. The wild‐type GSTA1‐1 C terminus explores a wide range of conformational space without significant barriers, whereas the GSTA4‐4 remains relatively immobile in a narrow conformational energy well. The mutants span the range between the wild‐type enzymes.

The catalytic activity of each of the mutants was also determined with a range of substrates that represented a significant extent of chemical space, and their promiscuity *J*‐values were determined. The results demonstrate that, across the series of wild‐type and GST mutants, the width of the enthalpy landscape increased and the height of barriers between enthalpy states decreased, as the *J*‐values increased. The results with these GSTs indicate that conformational flexibility, as measured by these thermodynamic parameters, correlates with substrate promiscuity within this single protein fold 86. Smooth and wide conformational landscapes of the substrate‐free states would likely facilitate the recruitment of a wide range of substrates.

#### Kinetic consequences of conformational flexibility

Kinetic properties of these GSTs further revealed mechanistic aspects of their promiscuity. Pre‐steady kinetic analysis of the metabolism of two different substrates with the two most promiscuous GST enzymes in the series indicated that a conformational relaxation of the substrate‐free enzyme is kinetically significant for some, but not all substrates. That is, different combinations of substrate and GST variant differentially yielded pre‐steady‐state kinetic lags or bursts 86, 87. CS is likely to occur with nearly all enzymes to some degree, but the kinetic lags and bursts that can result are expected only when the conformational changes are slow on the timescale of catalysis or when many sequential conformational steps are required to convert nonbinding conformations to binding conformations. Taken together, the kinetic results are best explained by the presence of conformational ensembles for these promiscuous enzymes in the absence of ligands, where different substrates select different conformations. Interestingly, analogous behavior was observed with promiscuous cytosolic EHs and slow conformational changes in the substrate‐free ensemble were invoked to model the results 88, 89.

**Figure 3:**
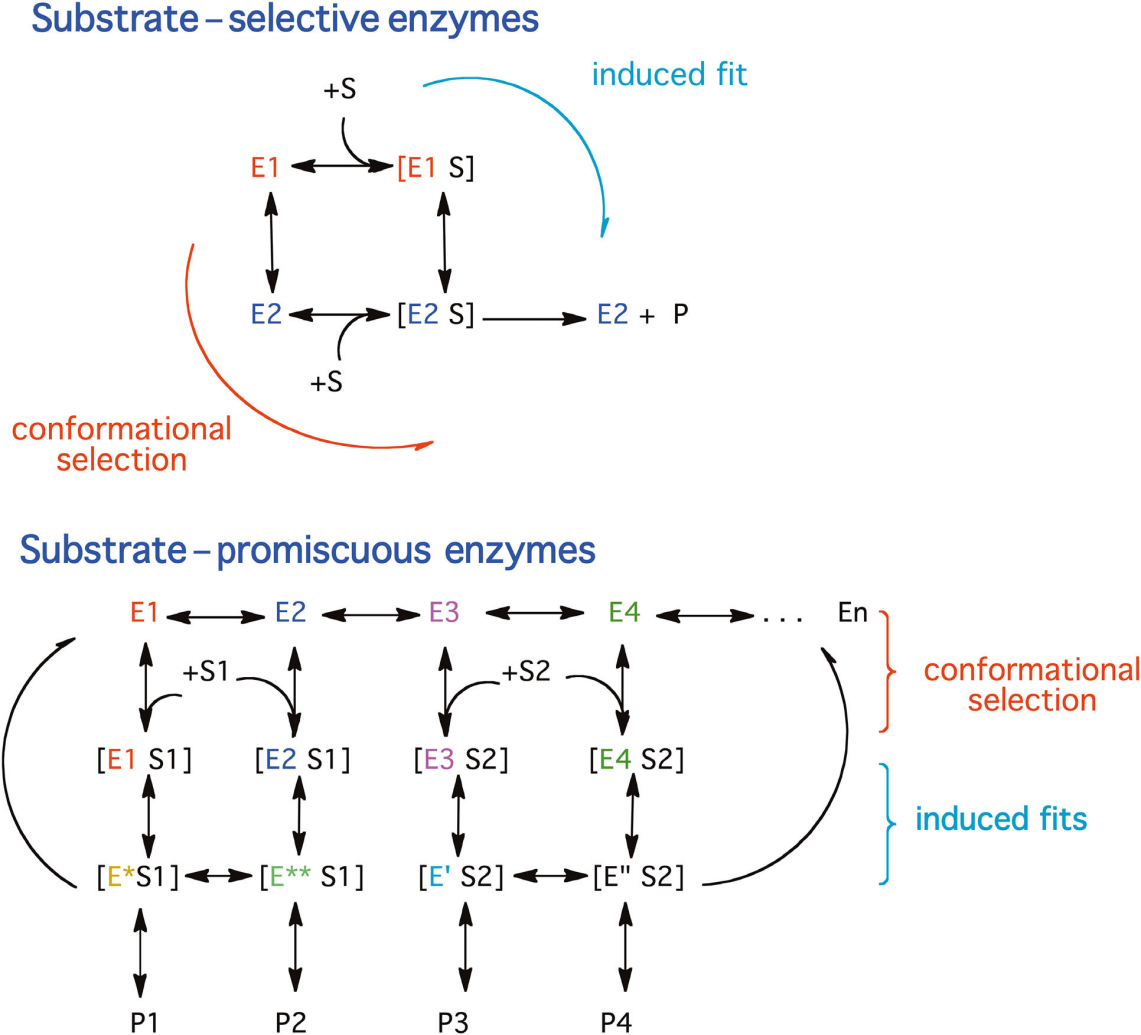
Kinetic models for substrate‐specific vs. substrate‐promiscuous enzymes. Top: Limiting case models for substrate‐specific enzymes are IF and CS. Most enzymes likely include a contribution of both, due to nominal conformational heterogeneity of the substrate‐free enzyme. Bottom: Proposed model for promiscuous drug metabolizing enzymes. Different conformations of enzyme are color‐coded. A wide range of conformational states of the substrate‐free enzyme (E1, E2… En) is presented to recruit structurally distinct substrates. Different substrates (S1, S2) bind to one or more conformations of the enzyme, indicated by the CS step, followed by conformational changes to the catalytically productive states that are retained at the active site (IF). Different substrates can induce different multiple conformations (E* vs. E** for S1 and E’ vs. E” for S2). Different complexes for the same substrate result in product promiscuity (P1 vs. P2 and P3 vs. P4) and multiple ES complexes for a single substrate. The model combines data from GSTs, CYPs, and other detoxication enzymes.

The results are consistent with a model wherein these promiscuous GSTs sample many conformations in the absence of substrate, and different substrates select different conformations. This presentation of multiple conformations with different ‘substrate selectivities’ increases the promiscuity. Upon substrate binding, the flexibility of the proteins is sufficient to allow each substrate to induce the fit required to form a productive catalytically active complex. This model is schematized and compared to IF and CS for substrate‐specific enzyme in Fig. 3. Thus, the combination of CS and IF optimizes the promiscuity, and a high degree of protein flexibility is required. CS provides a mechanism for recruitment of the widest range of substrates, and IF provides a mechanism for increasing *k*_cat_/*K*_M_ for any substrate that induces a fit via retention of bound substrate in the active site. Notably, this suggested behavior is supported by results with a nonenzymatic antibody. Antibodies are usually considered to be highly specific binders of their antigens, and they do not approach the degree of promiscuity exhibited by detoxication enzymes. However, behavior analogous to the proposed utility of CS in detoxication enzymes has been demonstrated by Tawfik *et al*., wherein a conformational ensemble of an antibody in the absence of antigen includes conformations that prefer a noncognate antigen, and this contributes to binding promiscuity 90. Both structural and kinetic methods indicated conformational heterogeneity with selection of different conformations by different antigens followed by IF. Interestingly, in the case of an antibody the ‘promiscuous’ binding could be a disadvantage if it induces an allergic response or advantageous if it expands the repertoire of a germline antibody. Similarly, catalytic antibodies with ester hydrolytic activity exhibit IF, CS, and kinetic lags associated with turnover‐dependent redistribution of the substrate‐free conformational ensemble 91.

These examples support the possible contribution of CS and IF to the complex kinetics observed with GSTA1‐1, and they indicate the role of CS in increasing the level of promiscuity. The interesting extension of these results is that detoxication enzymes have optimized these behaviors to further increase their substrate promiscuities.

#### Is this model supported for other drug metabolizing enzymes?

**Figure 4:**
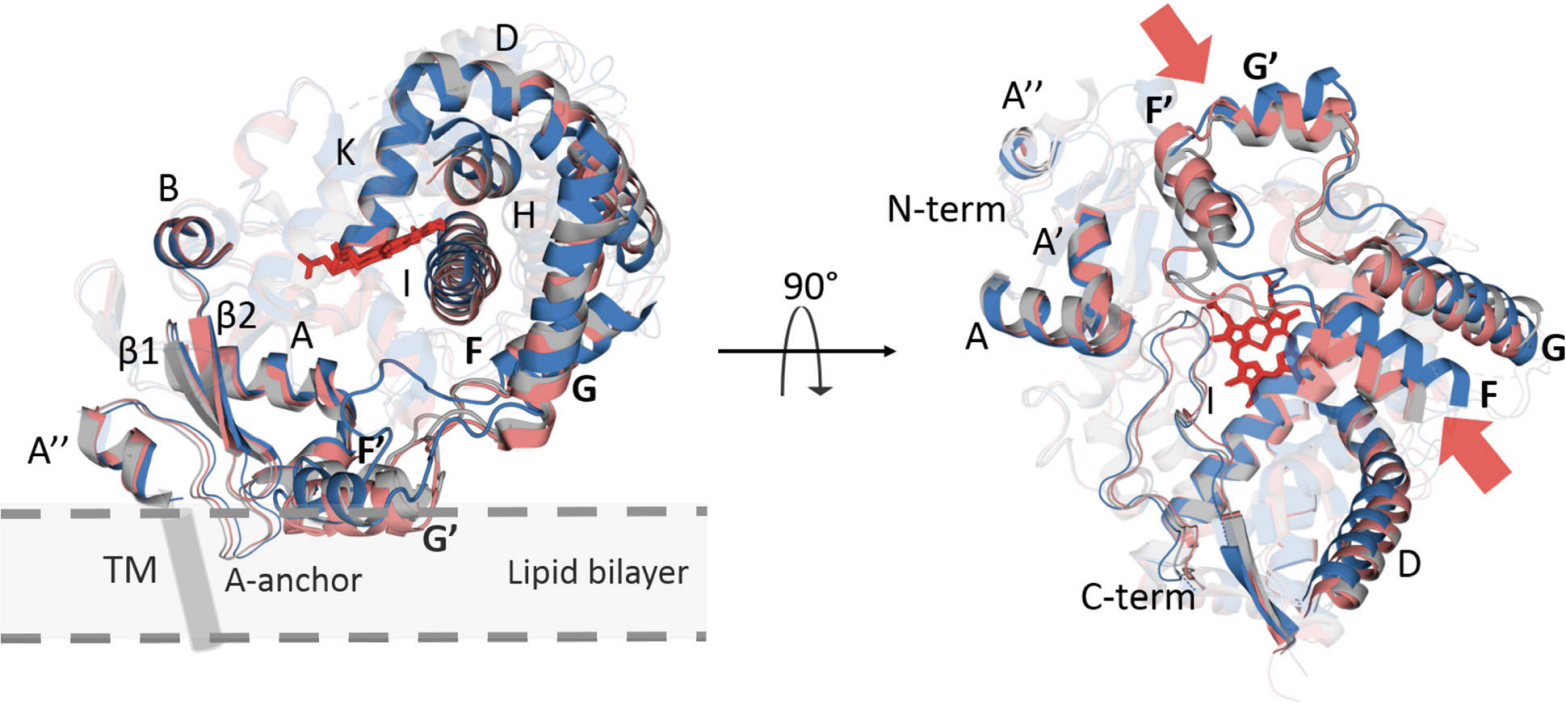
Superimposition of three substrate‐bound structures of CYP3A4: with midazolam (sky blue, PDB: http://www.rcsb.org/pdb/search/structidSearch.do?structureId=5TE8), ketoconazole (salmon, PDB: http://www.rcsb.org/pdb/search/structidSearch.do?structureId=2V0M), and ritonavir (gray, PDB: http://www.rcsb.org/pdb/search/structidSearch.do?structureId=5VC0). Each of the structures (chain A only) has been individually superimposed on the ligand‐free structure (not shown; PDB: http://www.rcsb.org/pdb/search/structidSearch.do?structureId=1TQN). Left: Three structures oriented to show the topology with respect to the membrane. The edge of the heme cofactor is observed (red). Substrates (not shown for clarity) sit below the heme toward the membrane and interact differentially with the D, E, F', F, G', G, H helices and the N‐term half of the I helix. Right: Rotation of the structures, viewed from the membrane. These elements occupy very different locations with different substrates resulting in large differences in active site volume when different ligands are bound. The most prominent differences occurred in the F’‐G’ and F‐G region (red arrows right panel). The active site and surroundings are highly plastic and adapt to different substrates.

Support for the contribution of the noncanonical type of IF to substrate promiscuity of drug metabolizing enzymes comes from numerous crystal structures, wherein different substrates bound to the same detoxication enzyme induce significantly different protein conformations. For example, CYP3A4 is the most promiscuous CYP isoform and different drugs or ligands induce structural changes that are widely distributed throughout the entire protein and different structural elements adopt ligand‐dependent locations and conformations 92, 93, 94, 95, 96. Moreover, large changes in the active site volume are observed when different ligands bind, consistent with a highly plastic overall fold that can adapt to ligands of different size. The estimated active site volume of CYP3A4 in the ligand‐free form vs. in the presence of ligands ketoconazole or erythromycin is 950Å^3^, 1650 Å^3^, and 2000 Å^3^, respectively 96. Such major changes in active site dimensions require significant plasticity. Interestingly, the conformational changes induced by substrates and inhibitors are globally distributed across large stretches of the entire structure. The largest amplitude conformational changes for CYP3A4 are observed near the presumed entrance to the active site, which is partially embedded in the membrane (Fig. 4). Many MD simulations support the overall theme that different substrates and ligands induce different conformational changes 97, 98, 99.

Recent kinetic data indicate that several promiscuous drug‐metabolizing CYPs and some substrate‐specific CYPs utilize CF 100. Included in this work are CYP–ligand combinations for which the ligand‐bound crystal structure is conformationally distinct from the ligand‐free CYP structure. Two scenarios are possible: 1) Multiple ligand‐free conformations are in equilibrium, and many ligands select a single or a set of related conformations and induce different ‘fits’; 2) the different ligands select different conformations from the ensemble that are like their bound conformations (no IF), but these different ligand‐free conformations are not represented in crystal structures. Presumably, the combination of IF and CF occurs. The fact that the kinetic experiments reveal CF indicates that the conformational exchange in the ligand‐free enzyme is slower than, or similar to, the bimolecular binding event. For the CYPs that demonstrate CF, the data were not analyzed in more detail so it has not been demonstrated directly that different ligands bind to different ligand‐free conformations of a single CYP. It is also noteworthy that neither the crystal structures nor the kinetic experiments were performed with a lipid bilayer present, and both structure and kinetics could be altered in a membrane.

**Figure 5:**
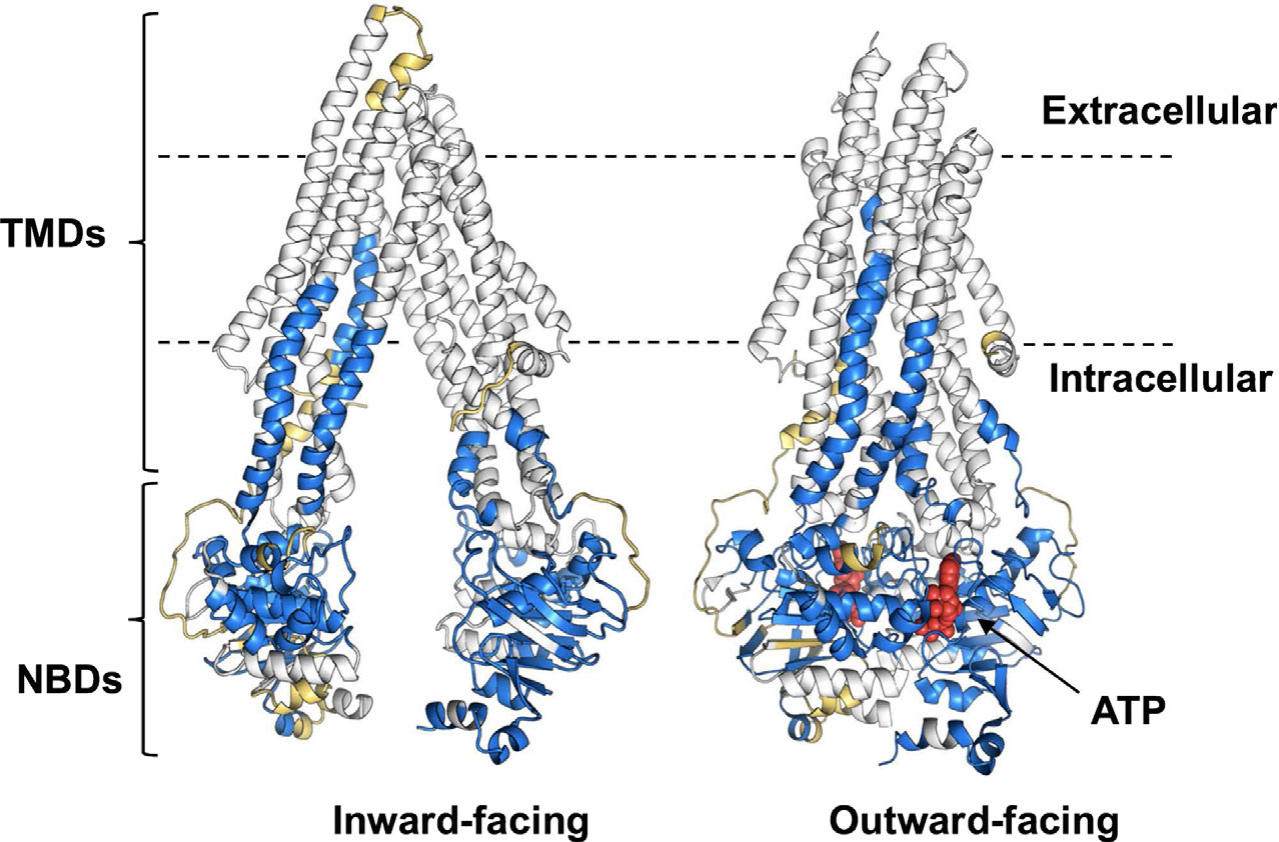
Extensive conformational heterogeneity of P‐gp revealed by H/DX MS. P‐gp consists of two intracellular nucleotide‐binding domains (NBDs) and twelve transmembrane helices that comprise the transmembrane domains that form the drug‐binding site. Two macroscopic conformations are well characterized. The inward‐facing form allows binding of drugs from the membrane, but they cannot diffuse to the extracellular side until the enzyme binds ATP (red) and switches to an outward‐facing conformation. H/DX MS studies 101 indicate that many peptides (blue) exhibit slow conformational exchange on a wide range of timescales. Ligand‐free P‐gp populates a wide range of conformations that likely increase its promiscuity via conformational selection. Yellow regions are peptides analyzed that do not exhibit slow conformational exchange.

**Figure 6:**
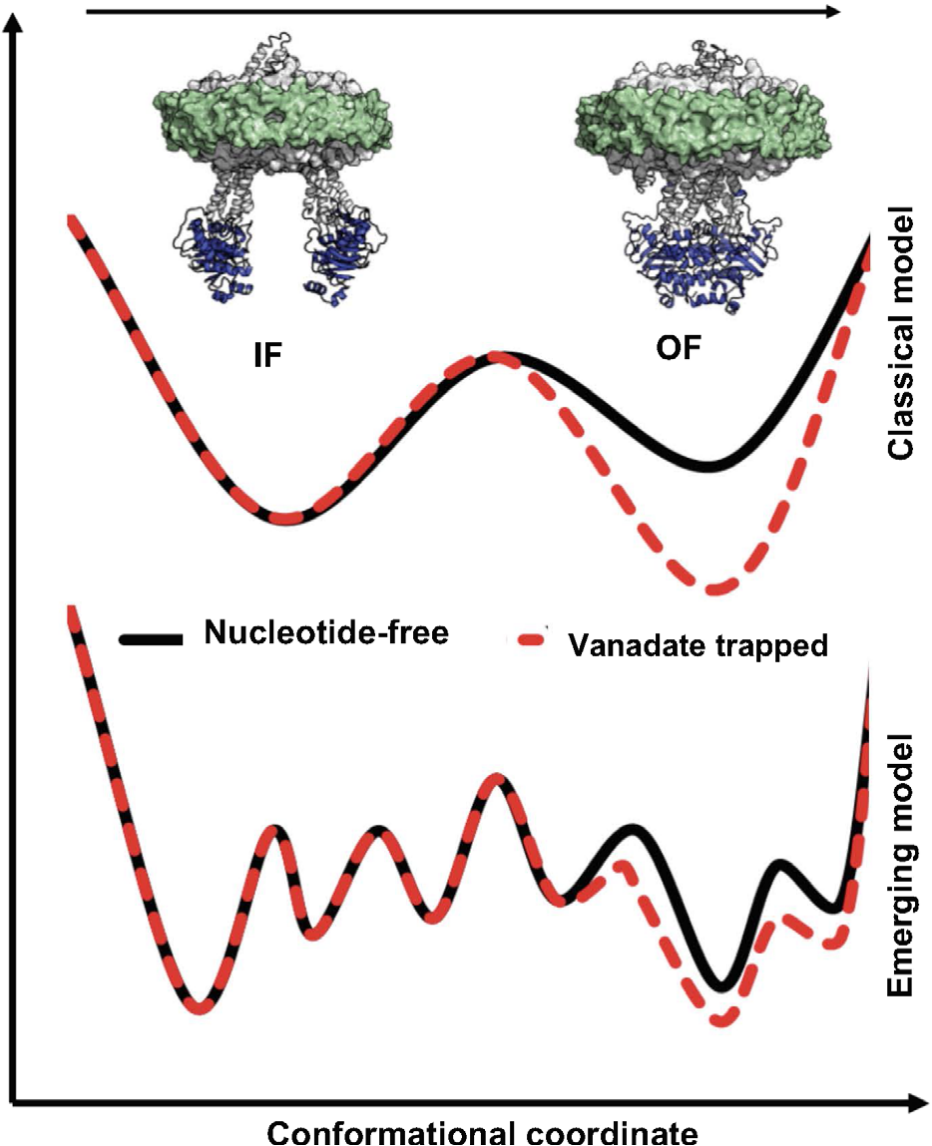
Schematized energy landscapes for P‐gp. The reaction coordinate for the nucleotide‐binding domain (NBD) moving together and apart is shown. Top: Simple two‐state model. The protein exists in inward‐facing (IF in the Figure, distinct from induced fit used elsewhere) and outward‐facing (OF) conformations, and nucleotide binding or ‘trapping’ shifts the ensemble partially to the OF state. Bottom: Several recent experimental methods suggest that there is significant conformational heterogeneity superimposed on IF and OF states. These additional conformations exchange on a wide range of timescales and present many conformations for substrates to select. The resulting energy landscape is rough, in contrast to that of GSTA1‐1. Both energy landscapes could be exploited to increase substrate promiscuity.

Regarding the potential contribution of CS to promiscuity, P‐gp presents a striking example of conformational heterogeneity in the substrate‐free enzyme. A few crystal structures of human and mouse P‐gp are available with drugs or substrates bound, so structural evidence for a wide range of substrate‐dependent conformation exists 68, 69, 70. Dynamic methods including time‐resolved AFM and H/D exchange mass spectrometry indicate interesting conformational changes on a wide range of timescales, from seconds to hours, for the ligand‐free enzyme that persists in the outward‐facing closed state 67, 101 (Fig. 5). Together, the data indicate a rough energy landscape in the absence of drugs with a heterogeneous ensemble of conformations exchanging on different timescales, and this is consistent with the role of CS in the substrate promiscuity of P‐gp (Fig. 6).

Importantly, the conformational heterogeneity of the substrate‐free P‐gp is distinctly different from GSTA1‐1, in as much as the energy landscape must include significant energy barriers to rearrangement, in contrast to the barrierless, fluid, and landscape of GSTA1‐1. Speculatively, if substrates for P‐gp do not drive the protein to a homogeneous ensemble on timescales similar to transport, then only a portion of the enzyme population would participate in the transport at early times during catalytic transport. With extended exposure to the same substrate, the entire P‐gp population might be engaged, as suggested by model studies for generic enzymes exhibiting slow conformational changes.

## Summary

**Figure 7:**
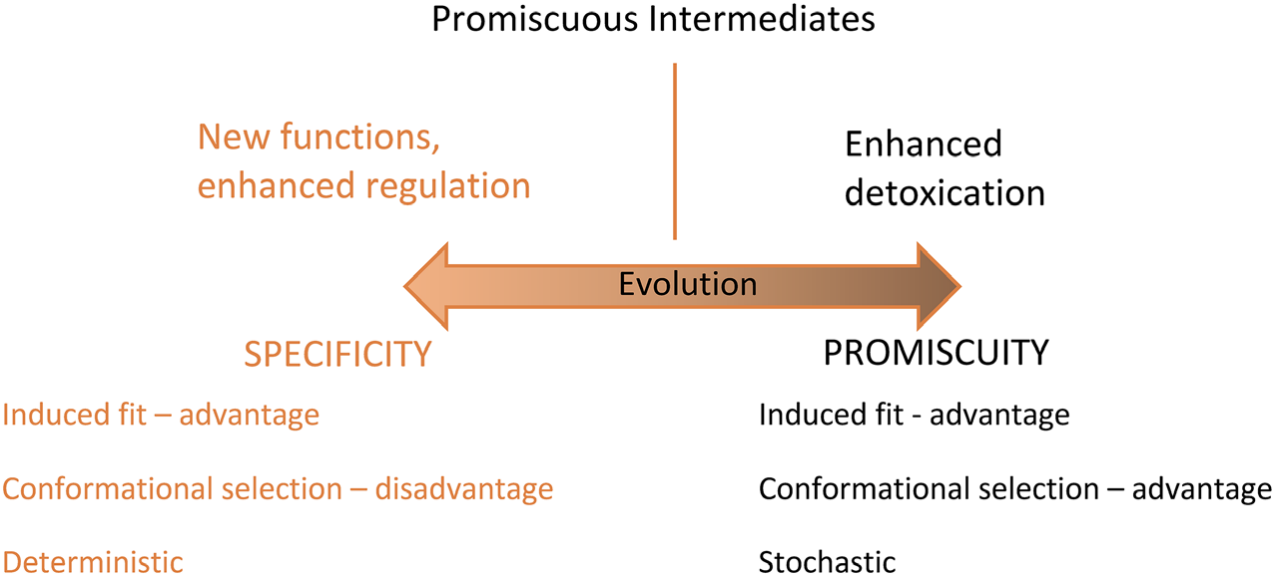
Comparative overview of the properties of substrate‐specific enzymes and promiscuous detoxication enzymes that are altered by evolution, and the differential exploitation of conformational selection.

Drug metabolizing enzymes are among the most substrate and catalytically promiscuous of any enzymes, with properties that contrast some of the properties of promiscuous enzymes that are formed from random mutations of substrate‐specific enzymes, as schematized in Fig. 7. The early suggestions of Jakoby are largely correct. Most, but not all, detoxication enzymes utilize highly reactive cofactors or cosubstrates that indiscriminately encounter substrates, contributing to both substrate and product promiscuity. The large and highly plastic active sites of drug metabolizing enzymes certainly contribute to the promiscuity of these enzymes. The plasticity may be due to smooth conformational landscapes with minimal barriers to rearrangement, as experimentally found for GSTA1‐1, or it may provide persistent conformational heterogeneity that corresponds to rough conformational landscapes with slowly equilibrating states, as suggested for P‐gp. Either case could lead to conformational selection that increases substrate promiscuity, and could be an advantage uniquely exploited to increase promiscuity, in contrast to substrate‐selective enzymes which gain no catalytic advantage from CS. The substrate promiscuity of the detoxication enzymes is likely to have been optimized by selective pressure, with combinations of reactive cofactors and exploitation of conformational ensembles in the absence of substrate that sample additional conformations upon substrate binding.

## Conflicts of interest

The authors declare no conflict of interest.
